# Brief Research Report: Virus-Specific Humoral Immunity at Admission Predicts the Development of Respiratory Failure in Unvaccinated SARS-CoV-2 Patients

**DOI:** 10.3389/fimmu.2022.878812

**Published:** 2022-04-25

**Authors:** Ana Tajuelo, Octavio Carretero, Estéfani García-Ríos, Mireia López-Siles, Olga Cano, Mónica Vázquez, Vicente Más, Isabel Rodríguez-Goncer, Antonio Lalueza, Francisco López-Medrano, Rafael San Juan, Mario Fernández-Ruiz, José Mᵃ Aguado, Michael J. McConnell, Pilar Pérez-Romero

**Affiliations:** ^1^ Intrahospital Infections Laboratory, National Centre for Microbiology, Instituto de Salud Carlos III (ISCIII), Madrid, Spain; ^2^ Unit of Infectious Diseases, Hospital Universitario “12 de Octubre”, Instituto de Investigación Sanitaria Hospital “12 de Octubre” (imas12), Madrid, Spain; ^3^ Infecciones Víricas e Inmunidad en Enfermos Inmunodeprimidos, National Centre for Microbiology, Instituto de Salud Carlos III (ISCIII), Madrid, Spain; ^4^ Universidad Internacional de Valencia – VIU, Valencia, Spain; ^5^ Biología Viral, National Centre for Microbiology, Instituto de Salud Carlos III (ISCIII), Madrid, Spain; ^6^ Department of Internal Medicine, Hospital Universitario “12 de Octubre”, Instituto de Investigación Sanitaria Hospital “12 de Octubre” (imas12), Madrid, Spain; ^7^ Centro de Investigación Biomédica en Red de Enfermedades Infecciosas (CIBERINFEC), Madrid, Spain; ^8^ Department of Medicine, Universidad Complutense, Madrid, Spain

**Keywords:** SARS-CoV-2, COVID disease severity, humoral response, IgG, IgM 2

## Abstract

**Introduction:**

There is robust evidence indicating that the SARS-CoV-2-specific humoral response is associated with protection against severe disease. However, relatively little data exist regarding how the humoral immune response at the time of hospital admission correlates with disease severity in unimmunized patients. Our goal was toidentify variables of the humoral response that could potentially serve as prognostic markers for COVID-19 progressionin unvaccinated SARS-CoV-2 patients.

**Methods:**

A prospective cross-sectional study was carried out in a cohort of 160 unimmunized, adult COVID-19 patients from the Hospital Universitario 12Octubre. Participants were classified into four clinical groups based on disease severity: non-survivors with respiratory failure (RF), RF survivors, patients requiring oxygen therapy and those not receiving oxygen therapy. Serum samples were taken on admission and IgM, IgG, IgG subclass antibody titers were determined by ELISA, and neutralizing antibody titersusing a surrogate neutralization assay. The differences in the antibody titers between groups and the association between the clinical and analytical characteristics of the patients and the antibody titers were analyzed.

**Results:**

Patients that developed RF and survived had IgM titers that were 2-fold higher than non-survivors (*p* = 0.001), higher levels of total IgG than those who developed RF and succumbed to infection (*p*< 0.001), and than patients who required oxygen therapy (*p*< 0.05), and had 5-fold higher IgG1 titers than RF non-survivors (*p*< 0.001) and those who needed oxygen therapy (*p*< 0.001), and 2-fold higher than patients that did not require oxygen therapy during admission (*p*< 0.05). In contrast, RF non-survivorshad the lowest neutralizing antibodylevels, which were significantly lower compared those with RF that survived (*p* = 0.03). A positive correlation was found between IgM, total IgG, IgG1 and IgG3 titers and neutralizing antibody titers in the total cohort (p ≤ 0.0036).

**Conclusions:**

We demonstrate that patients with RF that survived infection had significantly higher IgM, IgG, IgG1 and neutralizing titers compared to patients with RF that succumb to infection, suggesting that using humoral response variables could be used as a prognostic marker for guiding the clinical management of unimmunized patients admitted to the hospital for SARS-CoV-2 infection.

## Introduction

Although most cases of severe acute respiratory syndrome coronavirus 2 (SARS-CoV-2) infection are asymptomatic or manifest as mild disease, a significant proportion of patients develop severe disease, most commonly pneumonia that can progress to acute respiratory distress syndrome (ARDS) and organ failure (1, 2). Severe SARS-CoV-2 infection is associated with high mortality (3, 4), and advanced age, malegender, high blood pressure, diabetes, obesity and chronic lung disease have been associated with severe disease (5). Additionally, immunological and biochemical variables such as the total number of neutrophils and lymphocytes, interleukin-6 (IL-6), C reactive protein (CRP), lactate dehydrogenase, d-dimers or ferritin levels have been associated with respiratory failure and death (6, 7).

The host immune response is essential in determining the course of disease after SARS-CoV-2 infection by promoting viral clearance and resolution of infection (8). Most symptomatic patients develop virus-specific IgM and IgG within two weeks after the onset of symptoms (9–11). However, the magnitude of the response is heterogeneous depending on the characteristics of the patients and their clinical course (12–14). Previous results have shown a correlation between IgG titers and neutralizing antibody titers, which function by blocking the entry of the virus into host cells (15–17). Higher virus-specific IgM and IgG, and virus neutralizing antibody titers against SARS-CoV-2 have been reported in patients with severe symptoms (10, 18–22). In addition, it has been suggested that the timing of the appearance of these antibodies is an important factor that conditions disease progression and viral load (23, 24). Additionally, the presence of antibodies during the early phases of infection has been correlated with improved clinical outcomes (25) and protection against reinfection (26–31).

In the present study we assess anti-SARS-CoV-2 spike (S) protein antibodies (IgM, IgG, and IgG subclasses) and neutralizing antibody titers in serum obtained at the time of admission from a cohort of unvaccinated patients hospitalized for coronavirus disease 2019 (COVID-19). We characterize associations between these indicators of virus-specific humoral immunity at hospital admission and respiratory failure in order to identify potential prognostic markers for COVID-19 progression.

## Materials and Methods

### Patient Recruitment and Sample Collection

Within an institutional cohort of consecutive patients admitted at the University Hospital “12 de Octubre” (Madrid, Spain) for COVID-19 within the first wave (between March and April 2020) we carried out a “nested case-control study” randomly selecting four groups of similar size with four different outcomes [respiratory failure (S/N) and death (S/N)], in which a “single-point assessment at the time of hospital admission” of humoral immunity was performed.

Blood samples were collected at the time of hospital admission(up to five days after admission) and stored at -80 °C until analysis. Serum samples were analysed at the Spanish National Centre for Microbiology (Majadahonda, Spain). Socio-demographic, epidemiological and infection-related variables were recorded. The study was approved by the local Ethics Committee for Clinical Research and was conducted following the Declaration of Helsinki and the Guidelines for Good Clinical Practice.Based on the exceptional situation caused by the pandemic, patients agree to participate but no informed consent was obtained, which was approved by the local Ethics Committee for Clinical Research.

### Study Definitions

Respiratory failure (RF) was defined as the development of acute respiratory distress syndrome (ARDS), defined as a partial pressure of arterial oxygen/fraction of inspired oxygen [PaO_2_/FiO_2_ ratio] ≤200 mmHg (32) or the need for mechanical ventilation. Cardiovascular disease was defined as the presence of coronary heart disease, heart failure and/or stroke. Chronic lung disease was defined as the presence of chronic obstructive pulmonary disease or severe obstructive sleep apnea.

Cohort participants were classified into four clinical groups according to the WHO Ordinal Scale for Clinical Improvement COVID-19 criteria (33). The group of non-survivors with RF corresponded to score 8 (death), RF survivors corresponded to score 7-5 (ventilation and additional organ support or intubation or mechanical ventilation and non-invasive ventilation or high-flow oxygen), patients that required oxygen therapy corresponded to score 4 (oxygen by mask or nasal prongs) and the group of patients not receiving oxygen therapy corresponded to score 3 (hospitalized, no oxygen therapy).

Oxygen saturation was obtained from hospital admission and on room air.

Length of clinical course was defined as the elapse from the symptoms onset to the end of the clinical stay (hospital discharge or death). Length of hospital stay was defined as the elapsed time from the hospital admission to the end of the clinical stay (hospital discharge or death).

### Laboratory Measurements

CRP, IL-6, albumin, alanine transaminase (ALT), aspartate transaminase (AST), lactate dehydrogenase (LDH), ferritin, D-dimer, fibrinogen, triglycerides and procalcitonin levels, platelet, neutrophil, lymphocyte and monocyte counts were routinely measured during standard care. All laboratory measurements were performed on the same dayat admission.

### Enzyme-Linked Immunosorbent Assays

Recombinant S protein was produced by transient transfection of FreeStyle 293F cells (Thermo Fisher) using polyethylenimine and a plasmid coding for a HexaPro derived construct (34) that includes the D614G substitution. The S ectodomainwas purified from filtered cell supernatants using HisTrap™ Excel columns (CYTIVA) and subjected to an additional purification step by size-exclusion chromatography using a Superose 6 10/300 column (CYTIVA).

For indirect enzyme-linked immunosorbent assays (ELISAs) 96-well plates (Corning) were coated overnight with 100 ng per well of purified recombinant S protein in PBS. Unbound proteins were removed by washing twice with 200 µL of 0.1% Tween 20 in PBS (PBST). Blocking was performed with 100 µL of PBST supplemented with 5% skim milk (PBSTM) for 1 h. After the wells were washed twice as detailed above, serial two-fold dilutions of serum samples (from 1:100 to 1:204800) were added and incubated for 1 h at room temperature (RT), followed by three washing steps with PBST. Then, 100 µL of horseradish peroxidase-conjugated anti-human IgG (1:3000 dilution; 62-8420, Invitrogen), anti-human IgM (1:10000 dilution; A18841, Novex), anti-human IgG1 (1:1000 dilution; MH1715, Invitrogen), anti-human IgG2 (1:1000 dilution; MH 1722, Invitrogen), anti-human IgG3 (1:5000 dilution; MA5-16718, Invitrogen) or anti-human IgG4 (1:1000 dilution; A10654, Invitrogen) were added to each well and incubated for 1 h atRT. After four washing steps with PBST, 100 µL of horseradish peroxidase substrate (54827-17-7, Sigma-Aldrich) was added and incubated for 15 min at RT. The reaction was stopped using 50 µL 1 N sulfuric acid. Absorbance at 450 nm was measured using a plate reader (Tecan Sunrise). Titerswere defined as the highest dilution of serum at which the OD_450_ was at least 0.2 above background wells (wells without serum).

### ACE-2/S Protein Inhibition Assay

To evaluate the potential neutralizing activity of antibodies, S protein binding competition assays between the serum samples and a monomeric form of the human angiotensin converting enzyme 2 (ACE2) were performed. Briefly, microtiter 96-well plates were coated with a chimeric version of a monoclonal anti-Foldon antibody (35)overnight at 8 ng/µL in PBS. The next day, plates were blocked with PBS supplemented with BSA fraction V (Sigma) at 1% (PBS-BSA 1%). Then, purified Hexapro (34) derived construct containing the D614G substitution was captured by incubation at 1 ng/µL in PBS-BSA 1% during 45 min at RT. Following protein incubation, plates were washed with PBS, and successive incubations at RT of sera dilutions and hACE-2 monomeric-StreTag receptor (20 ng/µL) complexed with StrepTactin-HRP (1:5000) were performed. Sera incubation was prolonged for 45 min and after a 15 min incubation of receptor-StrepTactin HRP complexes, receptor binding to captured S protein was revealed with the OPD substrate (Sigma Aldrich), and the signal was measured in a spectrophotometer reading OD at 493-620nm. Assay background was determined in parallel plates with aSprotein locked in the closed conformation that is unable to bind the hACE-2 receptor. For this purpose, a purified HexaPro derived construct including a double cysteine substitution, S383C D985C, was used. A pool of sera from individuals negative for anti-SARS-CoV-2 antibodies and hACE-2 monomeric untagged receptor were used as negative and positive controls, respectively. After subtraction of the background, the percentage of neutralization was calculated as [1- (OD_495-620_ test serum/OD_495-620_ negative control)] x 100%. In all experiments incubation of hAE-2 untagged receptor at 200 ng/µL achieved a neutralization rate higher than 85%.

### Statistical Analyses

Patients were considered SARS-CoV-2 seropositive if antibody titers determined by ELISA were higher than 50 (limit of detection of the assay) and they were considered to have positive neutralizing antibodies if the titer was greater than 3.3 (36). Results were expressed as the mean ± standard deviation (SD) for continuous variables with parametric distribution or the median with interquartile range (IQR) for those with non-parametric distribution. Spearman’s correlation coefficients and *P*-values were calculated to evaluate the correlation between quantitative variables. Comparison of antibody titers between groups was performed using the Kruskal-Wallis test. Subsequent pairwise analyses between disease severity groups were performed using Dunn’s multiple comparisons test. Non-parametric statistical tests were used given the non-normal distribution of the data and the lack of homoscedasticity as assessed by Kolmogorov-Smirnov and Levene tests, respectively. Statistical analyses and data plotting were performed using Prism 5 v.5.01 (GraphPad Software) and SPSS (IBM). *P*-values ≤ 0.05 were considered statistically significant.

## Results

### Cohort Characteristics

Global characteristics of the four different clinical groups included in the study (RF non-survivors, RF survivors, oxygen therapy and non-oxygen therapy) are shown in **Table 1** and **Supplementary Table 1**. The median age of the cohort was 61 years (IQR: 49.25-75) with 68.8% (110/160) of male patients and 70% (112/160) of Caucasian race. The age of RF non-survivors was significantly higher compared to the other three clinical groups (*P*< 0.001). The most frequent comorbidities were hypertension (35.6%), dyslipidemia (27.5%), diabetes (20%), cardiovascular disease (15.6%) and chronic lung disease (6.3%). As expected, the group of RF non-survivors had a significantly higher proportion of patients with hypertension (*p* = 0.035), chronic lung disease (*p* = 0.006) and diabetes (*p* = 0.043) than the other clinical groups. This group also had significantly (*p* < 0.001) lower oxygen saturation compared to the other three groups, while the group of RF survivors had significantly higher levels of LDH, CRP, AST and leukocyte and neutrophil counts (*p* < 0.001). No significant differences were observed in days from symptom onset to hospital admission or the timing of sampling across each of the clinical groups.

**Table 1:** Demographic, clinical and analytical characteristics at hospital admission.

Characteristics	Total (N=160)	RF non-survivors (n=40)	RF survivors (n=40)	Oxygen therapy(Non RF) (n=40)	Non Oxygen therapy (n=40)	*p*-value
						
Age (y), median (IQR)	61 (49.25-75)	76.5 (68.75-83)	55 (47–65)	65 (48.75-75.75)	51.5 (43-57)	**<0.001**
Sex, male, no. (%)	110 (68.8%)	29 (72.5%)	30 (75%)	28 (70%)	23 (57.5%)	0.337
Race, no. (%)						0.287
Caucasian	112 (70%)	34 (85%)	22 (55%)	28 (70%)	28 (70%)	
Hispanic	43 (26.9%)	6 (15%)	15 (37.5%)	10 (25%)	12 (30%)	
Others	5 (3.1%)	0 (0%)	3 (7.5%)	2 (5%)	0 (0%)	
Comorbidities, no. (%)						
Hypertension	57 (35.6%)	19 (47.5%)	16 (40%)	15 (37.5%)	7 (17.5%)	**0.035**
Cardiovascular disease	25 (15.6%)	10 (25%)	5 (12.5%)	7 (17.5%)	3 (7.5%)	0.167
Chronic pneumopathy	10 (6.3%)	7 (17.5%)	1 (2.5%)	2 (5%)	0 (0%)	**0.006**
Diabetes	32 (20%)	13 (32.5%)	9 (22.5%)	7 (17.5%)	3 (7.5%)	**0.043**
Dyslipidemia	44 (27.5%)	15 (37.5%)	8 (20%)	11 (27.5%)	10 (25%)	0.353
Obesity *data not available in all cases	55 (43.3%) N=127	19 (57.6%)n=33	15 (46.9%)n=32	11 (34.4%)n=32	10 (33.3%)n=30	0.161
Smoking *data not available in all cases	11 (7.2%)N=152	2 (5.1%)n=39	4 (10%)n=40	3 (8.6%)n=35	2 (5.3%)n=38	0.795
Neoplasia	15 (9.4%)	6 (15%)	2 (5%)	4 (10%)	3 (7.5%)	0.462
Charlson comorbidity index, median (IQR)	2 (1-4)	4 (3-6)	2 (1-3)	2.5 (1-4)	1 (0-2)	**<0.001**
Analytical characteristics ***§***						
Oxygen saturation (%) *data not available in all cases	92 (88-96)N= 159	88 (83.25-90) n=40	90 (85-92)n= 39	94 (91-96)n=40	98 (96-99)n=40	**<0.001**
LDH level (U/L)	379.5 (304.5-493.3)	443 (377.75-590.25)	494 (392-621)	335 (303.75-389.5)	290 (236.5-372)	**<0.001**
CRP level (mg/dL)	10.93 (4.75-18.03)	15.44 (10.74-23.57)	18 (9.87-25.53)	8.86 (3.9-12.34)	3.73 (1.62-7.66)	**<0.001**
ALT level (U/L) *data not available in all cases	32 (21-50)N=159	30.5 (20-45.8) n=40	42 (28-70.3)n=40	29 (20.3-52.5)n=40	29 (17-52)n=39	0.07
AST level (U/L)	42.5 (30-62.8)	51 (37.8-70)	53 (39.3-74.5)	35.5 (30-59)	28 (24-47.5)	**<0.001**
Albumin level (g/dL) *data not available in all cases	3.8 (3.4-4.1) N=151	3.6 (3.3-3.9)n=39	3.6 (3.3-4.0)n=38	3.9 (3.6-4.2)n=37	4.1 (3.8-4.4)n=37	**<0.001**
Ferritin level (ng/mL) *data not available in all cases	990 (508-1792) N=143	1532 (739-2254)n=35	1664 (978.3-2096.7) n=34	784 (449.5-1422.5) n=36	522.9 (201.3-840.3)n=38	**<0.001**
Leucocyte count (x 10^3^ cells/µL)	6.1 (4.8-7.7)	6.1 (4.6-8.8)	7.2 (5.7-11.1)	5.8 (4.3-7.1)	5.4 (4.5-6.5)	**<0.001**
Neutrophil count (x 10^3^ cells/µL)	4.5 (3.3-6.2)	5 (3.4-7.4)	5.9 (4.4-9.5)	4.2 (2.9-5.8)	3.8 (2.7-4.5)	**<0.001**
Lymphocyte count (x 10^3^ cells/µL)	0.8 (0.6-1.2)	0.7 (0.6-1.05)	0.8 (0.6-1.1)	0.9 (0.53-1.2)	1 (0.7-1.38)	0.064
COVID-19 treatment on serum extraction, no. (%)						
Hydroxychloroquine	152 (95%)	37 (92.5%)	40 (100%)	38 (95%)	37 (92.5%)	0.368
Lopinavir/ritonavir *data not available in all cases	109 (85.8%) n= 127	27 (84.4%) n=32	30 (96.8%) n=31	24 (82.8%) n=29	28 (80%) n=35	0.230
IFN-b *data not available in all cases	32 (20.3%) n= 158	16 (40%) n=40	12 (30.8%) n=39	2 (5.1%) n=39	2 (5%) n=40	**<0.001**
Tocilizumab	36 (22.5%)	8 (20%)	19 (47.5%)	7 (17.5%)	2 (5%)	**<0.001**
Corticoesteroids	76 (47.5%)	26 (65%)	31 (77.5%)	14 (35%)	5 (12.5%)	**<0.001**
Time from onset of symptoms to hospital admission (d), median (IQR)	6 (4-8)	6 (4.25-8)	6 (4-7)	6 (5-8)	6.5 (4-9.75)	0.885
Time from onset of symptoms to serum extraction (d), median (IQR)	9 (7-11)	9 (6.25-11)	8 (7-10)	9 (7-10)	9 (6-11.75)	0.970
**Length of clinical course (d), median (IQR)**	**17 (12-25)**	**14 (11-20)**	**27.5 (22-38.5)**	**17 (12.25-24.75)**	**13 (9.25-16)**	**<0.001**

RF, respiratory failure. **§** represented median and interquartile range.

Bold values indicate stastisticaly signifcant.

### Humoral Response and Disease Severity

**Figure 1:**
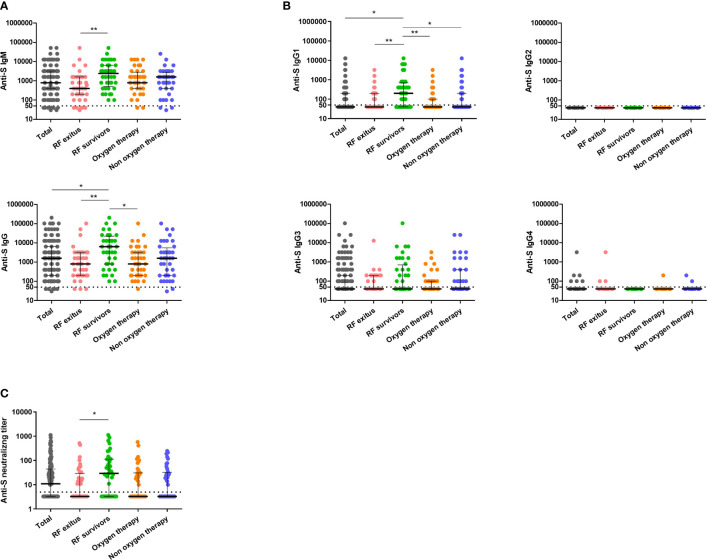
Analysis of antibody levels and neutralizing activity against SARS-CoV-2 S protein in serum samples from COVID-19 patients. Anti-S IgM, anti-S IgG **(A)** anti-S IgG1, anti-S IgG2, anti-S IgG3 and anti-S IgG4 **(B)** and neutralizing titers **(C)** are indicated for the total cohort of patients (grey circles, n = 160) and the different disease severity groups: RF non-survivors (pink circles, n = 40), RF survivors (green circles, n = 40), oxygen therapy (orange circles, n = 40) and non-oxygen therapy (blue circles, n = 40). Cutoff value to determine positive (above) and negative (below dashed line) samples is indicated. Black lines represent medians and interquartile range. Statistical significance was determined by the non-parametric Kruskall-Wallis and Dunn’s Multiple Comparison tests, where *p< 0.05 and **p< 0.001. RF indicates respiratory failure. Y axis is represented by logarithmic scale.

The magnitude of the humoral response at the time of admission was characterized for all patients by determining endpoint titers for SARS-CoV-2 IgM and total IgG and IgG subclasses (**Figure 1**). IgM titers ranged from 100 to 51200, and eleven samples had IgM titers below the limit of detection. Patients that developed RF and survived had IgM titers 2-fold higher than non-survivors (*p* = 0.001), whereas no significant differences in IgM titers were observed when compared with other subgroups. Patients that developed RF and survived also had higher levels of total IgG than those who developed RF and succumbed to infection (*p* < 0.001), and that patients who required oxygen therapy (*p* < 0.05). IgG titers ranged from 100 to 204800 and six patients had titers below the limit of detection at admission.

Regarding IgG subclasses, 44.38% of patients had detectable levels of IgG1, and 38.75% had detectable levels of IgG3. While no significant differences were found in the IgG3 titers between groups, the group of RF survivors had 5-fold higher IgG1 titers than RF non-survivors (*p* < 0.001) and those who needed oxygen therapy (*p* < 0.001), and 2-fold higher than patients that did not require oxygen therapy during admission (*p* < 0.05).

Surrogate neutralization assays were performed in order to characterize the functional activity of the antibodies present in patient samples at the time of admission. Neutralizing antibodies were detected in 51.9% of patients, with titers ranging from 10 to 1115 (**Figure 1**). Subjects within the group of RF non-survivors had the lowest level of neutralizing antibodies, that were significantly lower compared with those with RF that survived (*p* = 0.03).

**Figure 2:**
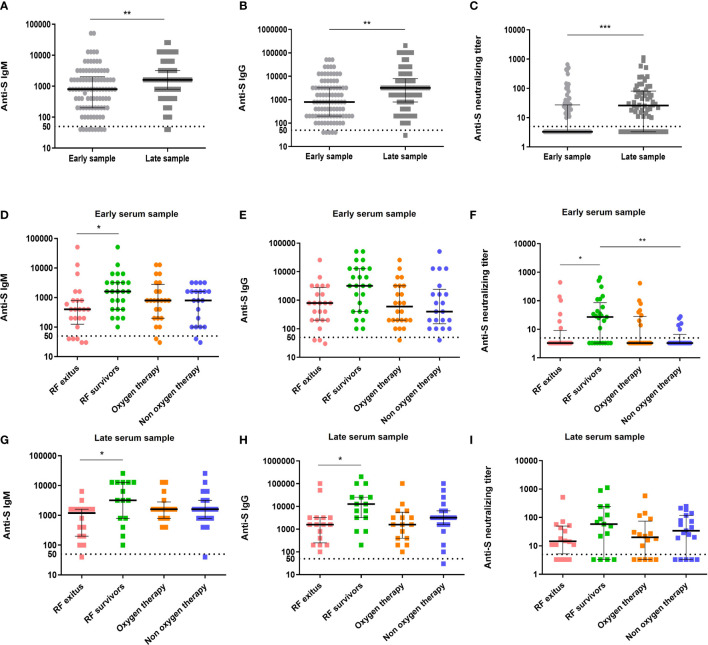
Antibody levels and neutralizing activity against SARS-CoV-2 by sample precocity in total cohort and by clinical group. Anti-S IgM, anti-S IgG and neutralizing titers were determined in early serum samples (ES, collected in the first nine days of symptoms) and in late serum samples (LS, collected from ten days of symptoms) for the total cohort **(A–C)** (light grey circles, n = 94 and dark grey squares, n = 66, respectively) and for the different disease severity groups **(D–I)** RF non-survivors (pink circles, ES: n = 24; pink squares, LS: n = 16), RF survivors (green circles, ES: n = 25; green squares, LS: n = 15), oxygen therapy (orange circles, ES: n = 24; orange squares, LS: n = 16) and non-oxygen therapy (blue circles, ES: n = 21; blue squares, LS: n = 19). Cutoff value to determine positive (above) and negative (below dashed line) samples is indicated and black lines represent medians and interquartile range. Statistical analysis were performed using Mann-Whitney U test and Kruskall-Wallis and Dunn’s Multiple Comparison tests. *p< 0.05 **p< 0.01 and ***p< 0.001. RF indicates respiratory failure. Y axis is represented by logarithmic scale.

Our analysis also allowed us to determine the effect of time from symptom onset on antibody and neutralizing titers. Serum samples from patients were extracted at hospital admission, but within a variable number of days from self-reported symptom onset. In order to characterize the effect of time from symptom onset to antibody levels, antibody levels and neutralizing activity were analyzed between patients with samples collected within the first 9 days of symptoms (early serum sampling, n = 94) and those whose samples were collected from ten days of symptoms (late serum sampling, n = 66). In the total cohort, IgM and IgG titers were significantly higher in patients with late serum sampling (*p* < 0.01) (**Figure 2**). When considering the different clinical groups, RF survivors had higher IgM titers than non-survivors (*p <*0.05), regardless the timing of sampling. However, when analyzing IgG titers, this significant difference was also observed in patients with late (*p* < 0.05) but not with early serum sampling (**Figure 2**). Regarding neutralizing activity, 37.2% of patients with early serum sampling presented neutralizing antibodies and the titer was lower than in patients with late serum sampling (72.7% *p* < 0.001) (**Figure 2**).

### Correlation Between Antibody Response and Neutralizing Activity by Disease Severity

Correlation between IgM, total IgG, IgG1 and IgG3 titers and neutralizing antibody titers was assessed in the total cohort and in each disease group (**Supplementary Figure 1**), and in all cases a positive and significant correlation was found. However, differences in the correlation coefficient were observed. Specifically, the highest coefficient between IgM and neutralizing antibodies titers was found in the group of RF non-survivors (ρ = 0.7287). In contrast, the highest correlation coefficient between total IgG and neutralizing antibodies titers was observed in the group that required oxygen therapy (ρ = 0.5625). Regarding IgG subclasses, the highest correlation coefficient between neutralizing antibodies and IgG3 titers was also found in the group that required oxygen therapy (ρ = 0.6182), whereas for IgG1 and neutralizing antibodies titers the highest correlation coefficient was in the group of RF survivors (ρ = 0.6218).

### Clinical Variables Associated With a Neutralizing Antibody Response

The most meaningful correlations with neutralizing titer were observed for IgM titer (ρ = 0.68; *p* < 0.001) and IgG titer (ρ = 0.65; *p* < 0.001), specifically IgG1 titer (ρ = 0.64; *p* < 0.001) and IgG3 titer (ρ = 0.61; *p* < 0.001). Neutralizing titers also correlated with oxygen saturation (ρ = -0.20; *p* = 0.013), LDH (ρ = 0.28; *p* < 0.001), CRP (ρ = 0.33; *p* < 0.001), leucocyte count (ρ = 0.35; *p* < 0.001), neutrophil count (ρ = 0.39; *p* < 0.001), platelet count (ρ = 0.27; *p* < 0.001), ALT (ρ = 0.19; *p* = 0.014), procalcitonin (ρ = 0.31; *p* = 0.015), ferritin (ρ = 0.21; *p* = 0.014), fibrinogen (ρ = 0.41; *p* < 0.001) and D-dimers (ρ = 0.21; *p* = 0.034). In addition, patients who received tocilizumab had higher neutralizing titers than those who did not [28 (IQR: 0-113.5) vs 0 (IQR: 0-33.3); *p* = 0.012]. There was no correlation between neutralizing antibody titers and days from admission or the length of invasive mechanical ventilation (IMV) or intensive care unit (ICU) admission. In patients with early serum sampling, the variables with the highest correlation with neutralizing titer continued to be IgM (ρ = 0.67; *p* < 0.001) and IgG (ρ = 0.65; *p* < 0.001), and within this, IgG1 (ρ = 0.66; *p* < 0.001) and IgG3 (ρ = 0.58; *p* < 0.001) titers.

We performed a multivariate analysis including those variables that had significant association with neutralizing antibody titers in the univariate analysis (**Supplementary Table 2**). We found that IgM (B = 0.012; (95% CI: 0.009-0.015) *p <*0.001) and IgG1 titer (B = 0.02; (95% CI: 0.008-0.032) *p* = 0.002) were the variables that best predicted neutralizing antibody titer.In the case of patients with early samples, IgM titer (B = 0.009; (95% CI: 0.007-0.011) *p <*0.001) and IgG1 titer (B = 0.033; (95% CI: 0.013-0.052) *p* = 0.001)were the best predictors of neutralizing antibody titer.

### Correlation Between the Humoral Response and Age, Time Since Admission and Symptom Onset

To better understand the dynamics of the humoral response, total IgM and IgG levels were correlated with age, days since admission and time from symptom onset (**Supplementary Figure 2**). When considering the total cohort, a negative correlation was observed between both IgM and IgG and age (*p* = 0.0004 and *p* = 0.0462, respectively). This negative association was maintained only for IgM in the group that required oxygen therapy (*p* = 0.030). IgM and IgG titers showed a positive correlation with days from admission (*p* = 0.0008 and *p* = 0.0014, respectively) and from symptom onset (*p* < 0.0001 and *p* < 0.0001, respectively) when considering the total cohort. Interestingly, patients with RF featured a positive correlation between both IgM and IgG titers during the first five days since admission, regardless of the clinical endpoint (survivors or non-survivors). A positive correlation between IgM (*p* = 0.0009) and IgG (*p* = 0.028) levels and days after symptom onset was observed for patients that required oxygen therapy. In addition, a positive correlation between IgG levels and time post-symptom onset was observed in subjects that did not require oxygen therapy (*p* = 0.007).

## Discussion

In the present study we characterized the virus-specific humoral response at the time of hospitalization in unvaccinated SARS-CoV-2 patients that presented different disease severities. Our results demonstrate that patients experiencing RF that survive infection have significantly higher anti-S protein IgM and IgG titers at the time of hospital admission compared to patients with RF that succumb to COVID-19. Additionally, non-survivors with RF demonstrated significantly lower neutralizing titers compared to patients with respiratory failure that survived. Taken together, these results indicate a protective role for S-protein specific and neutralizing antibodies in SARS-CoV-2 patients with respiratory failure, and is in line with findings from previous studies (26, 28–31, 37–39). Interestingly, patients experiencing RF that survived infection also demonstrated significantly higher anti-S protein IgG, but not IgM or neutralizing antibodies, compared to hospitalized patients requiring only oxygen therapy.This result may seem paradoxical in that patients with more severe disease demonstrated higher IgG titers than patients with less severe infections. However, similar results were found in a recent study by Yates et al. (8), in which unvaccinated patients with more severe disease had higher virus-specific and neutralizing antibody titers than patients with less severe infection (8).These results, as well as the results from our study, may suggest that patients experiencing more invasive disease mount a more robust humoral response to infection. These results should also be interpreted in the context of COVID-19 disease phase. The early appearance of IgM in the cohort groups likely corresponds to the viral phase, and these antibodies may play an important role in virus clearance. The latter IgG predominant response may be due to systemic inflammation and advanced disease.

Although there is robust evidence indicating that a humoral response directed against the SARS-CoV-2 S-protein, acquired either through vaccination or natural infection, is protective against severe infection (40, 41), there is only limited data regarding the association between S-protein specific IgG subclasses after infection and disease severity in unimmunized hospitalized patients (42). Furthermore, information regarding how IgG subclasses at the time of hospital admission correlate with disease severity is limited. In this study, we demonstrate that IgG1 and IgG3 are the predominant IgG subclasses produced after viral infection in all disease severity groups. This is in line with recent reports indicating that these IgG subclasses predominate after natural infection with SARS-CoV-2 (8, 43, 44), and previous reports indicating that these subclasses predominate after viral infections (45). Importantly, these previous studies also demonstrate very low titers of virus-specific IgG2 and IgG4 (8, 43, 44), similar to our results in the present study. A separate study demonstrated a predominance of IgG1 in healthcare workers after natural infection, similar to our results, but indicated that IgG2 titers were the second highest, followed by IgG3 and IgG4 (46). These differences may be due to differences in characteristics of the cohorts (hospitalized patients vs. healthcare workers) and in disease severity.

Our study also allowed us to assess associations between IgG subclasses and disease severity and neutralizing antibody titers in this patient population. Our results indicate that, similar to what was observed with total anti-S protein antibody titers, IgG1 titers were higher in patients with RF that survived infection compared to patients with RF that succumbed to infection and patients receiving oxygen therapy. In contrast to what was observed with total IgG, IgG1 titers were also significantly higher in patients with RF that survived infection compared to hospitalized patients that did not receive oxygen therapy. There were no other significant differences for the other IgG subclasses between diseases severity groups, although it is important to note that for IgG2 and IgG4 there was not a sufficient number of patients with detectable titers to perform a robust analysis. With respect to the correlation between anti-S protein antibody titers and neutralizing titers, positive correlations were observed for IgM, IgG, IgG1 and IgG3 in samples taken at the time of admission. These findings are in line with previous studies indicating that antibodies against the S protein correlate well with neutralization (47–50).

One limitation of this study is that in a small percentage of patients the serum sample was not collected on the same day of admission and the association of antibody titers with the analytical parameters may not be exact. In addition, clinical groups were not established as a function of age,which is a well-established as amajor risk factor for mortality in COVID-19.And finally, another limitation is that this study was carried out before the widespread dissemination of variants of concern that currently produce most infections globally, including the delta and omicron variants. The humoral response to these variants may be different than those assessed in this study, and resulting associations between disease severity and different variables of the immune response such as IgG, IgM and neutralizing titers may be different in unimmunized patients admitted to the hospital setting with infections caused by these variants. Further study is warranted in this area.

In conclusion, this study establishes correlations between COVID-19 severity and different variables of the humoral immune response at the time of hospital admission in unvaccinated patients. Our finding demonstrate that patients with RF that survived infection demonstrated significantly higher IgM, IgG, IgG1 and neutralizing titers compared to patients with RF that succumb to infection, suggesting that the humoral response plays a critical role in these patients, and raise the possibility of using humoral response variables as prognostic markers for guiding the clinical management of unimmunized patients admitted to the hospital for SARS-CoV-2 infection.

## Data Availability Statement

The original contributions presented in the study are included in the article/**Supplementary Material**. Further inquiries can be directed to the corresponding author.

## Ethics Statement

The studies involving human participants were reviewed and approved by Ethics committee for Clinical Investigation Hospital 12 de Octubre. Code: 20/269 Date:26/05/2020. The patients/participants provided their written informed consent to participate in this study.

## Author Contributions

IR-G, AL, FL-M, RJ, MF-R, JA: Provided patient care, selected the patients for the study and recruited samples, revised final version of the manuscript. JA : Study supervision, obtained funding, revised final version of the manuscript. AT: Performed ELISA assays and results analysis, manuscript draft. OCar: Samples recruitment and processing, clinical data analysis, neutralization assays and manuscript draft. EG-R: Surrogate neutralization assays optimization, results analysis support, manuscript draft. ML-S: Assistance in ELISA assays and results analysis, manuscript draft. VM, OCan, MV: Assistance in ELISA and neutralization assays, revised final version of the manuscript. MM: Conceived and supervised the study, drafted the manuscript. PP-R: Conceived and supervised the study, drafted the manuscript, obtained funding

## Funding

This work was supported by Mutua Madrileña Foundation (2020/0056) “Plan Nacional de I+D+I” and Instituto de Salud Carlos III (COVID-19 Research Call COV20/00181 and COV20_00679), Subdirección General de Redes y Centros de Investigación Cooperativa, Spanish Ministry of Science and Innovation, Spanish Network for Research in Infectious Diseases (REIPI RD16/0016) - co-financed by the European Development Regional Fund (EDRF) and the European Social Fund (ESF) "A way to achieve Europe- The ESF invests in your future". Red de Enfermedades Infecciosas (CIBERINFEC), CB21/13/00079. EG-R is supported by the Sara Borrell Program (CD18CIII/00007), MLS is supported by the Sara Borrell Program (CD17CIIII/00017), Instituto de Salud Carlos III, Ministerio de Ciencia, Innovación y Universidades., and AT is supported by the Garantía Juvenil Program of the Comunidad Autonoma de Madrid. IRG holds a research training contract “Río Hortega” (CM19/00163) and MFR a research contract “Miguel Servet” (CP18/00073), both from the Instituto de Salud Carlos III, Spanish Ministry of Science and Innovation.

## Conflict of Interest

MJM and PPR are founders and stockholders of the biotech-nology spin-off company Vaxdyn, which develops vaccines for infections caused by MDR bacteria. Vaxdyn had no role in the elaboration of this manuscript.

The remaining authors declare that the research was conducted in the absence of any commercial or financial relationships that could be construed as a potential conflict of interest.

## Publisher’s Note

All claims expressed in this article are solely those of the authors and do not necessarily represent those of their affiliated organizations, or those of the publisher, the editors and the reviewers. Any product that may be evaluated in this article, or claim that may be made by its manufacturer, is not guaranteed or endorsed by the publisher.
