# Corrigendum: Brief Research Report: Virus-Specific Humoral Immunity at Admission Predicts the Development of Respiratory Failure in Unvaccinated SARS-CoV-2 Patients

**DOI:** 10.3389/fimmu.2022.965809

**Published:** 2022-06-30

**Authors:** Ana Tajuelo, Octavio Carretero, Estéfani García-Ríos, Mireia López-Siles, Olga Cano, Mónica Vázquez, Vicente Más, Isabel Rodríguez-Goncer, Antonio Lalueza, Francisco López-Medrano, Rafael San Juan, Mario Fernández-Ruiz, José M. Aguado, Michael J. McConnell, Pilar Pérez-Romero

**Affiliations:** ^1^Intrahospital Infections Laboratory, National Centre for Microbiology, Instituto de Salud Carlos III (ISCIII), Madrid, Spain; ^2^Unit of Infectious Diseases, Hospital Universitario “12 de Octubre”, Instituto de Investigación Sanitaria Hospital “12 de Octubre” (imas12), Madrid, Spain; ^3^Infecciones Víricas e Inmunidad en Enfermos Inmunodeprimidos, National Centre for Microbiology, Instituto de Salud Carlos III (ISCIII), Madrid, Spain; ^4^Universidad Internacional de Valencia-VIU, Valencia, Spain; ^5^Biología Viral, National Centre for Microbiology, Instituto de Salud Carlos III (ISCIII), Madrid, Spain; ^6^Department of Internal Medicine, Hospital Universitario “12 de Octubre”, Instituto de Investigación Sanitaria Hospital “12 de Octubre” (imas12), Madrid, Spain; ^7^Centro de Investigación Biomédica en Red de Enfermedades Infecciosas (CIBERINFEC), Madrid, Spain; ^8^Department of Medicine, Universidad Complutense, Madrid, Spain

**Keywords:** SARS-CoV-2, COVID disease severity, humoral response, IgG, IgM 2

In the published article, the initials for an author in the article citation were incorrectly spelt as “Juan RS”. The correct spelling is “San Juan R”.

The authors apologize for this error and state that this does not change the scientific conclusions of the article in any way. The original article has been updated.

## Publisher’s Note

All claims expressed in this article are solely those of the authors and do not necessarily represent those of their affiliated organizations, or those of the publisher, the editors and the reviewers. Any product that may be evaluated in this article, or claim that may be made by its manufacturer, is not guaranteed or endorsed by the publisher.

