# Supplementary material for: Brief Research Report: Virus-Specific Humoral Immunity at Admission Predicts the Development of Respiratory Failure in Unvaccinated SARS-CoV-2 Patients
Source: Front Immunol. 2022 Apr 25;13:878812. doi: 10.3389/fimmu.2022.878812 (PMC9082065; doi:10.3389/fimmu.2022.878812)

**SUPPLEMENTAL MATERIALS**

**Supplementary Table 1**. Demographic, clinical and analytical characteristics at hospital admission.

| Characteristics | Total (N=160) | RF non-survivors (n=40) | RF survivors (n=40) | Oxygen therapy  (Non RF) (n=40) | Non Oxygen therapy (n=40) | *p* value |
| --- | --- | --- | --- | --- | --- | --- |
|  |  |  |  |  |  |  |
| Ethnicity Others, no. (%) |  |  |  |  |  | 0.287 |
| Subsaharian | 1 (0.6%) | 0 (0%) | 1 (2.5%) | 0 (0%) | 0 (0%) |  |
| Magreb | 2 (1.3%) | 0 (0%) | 1 (2.5%) | 1 (2.5%) | 0 (0%) |  |
| Asian | 1 (0.6%) | 0 (0%) | 0 (0%) | 1 (2.5%) | 0 (0%) |  |
| Gipsy | 1 (0.6%) | 0 (0%) | 1 (2.5%) | 0 (0%) | 0 (0%) |  |
| Comorbidities, no. (%) |  |  |  |  |  |  |
| Asthma | 9 (5.6%) | 1 (2.5%) | 3 (7.5%) | 2 (5%) | 3 (7.5%) | 0.730 |
| Transplant | 1 (0.6%) | 0 (0%) | 0 (0%) | 0 (0%) | 2 (2.5%) | 0.389 |
| Analytical characteristics ***§*** |  |  |  |  |  |  |
| IL-6 level (pg/mL)  *data not available in all cases | 38 (7.8-159.8)  N=16 | 79  n=1 | 166 (117-346)  n= 7 | 16 (2-31.5)  n=4 | 10 (2.3-35.8)  n=4 | **0.03** |
| Procalcitonin level (ng/mL) *data not available in all cases | 0.14 (0.09-0.42) N=63 | 0.38 (0.19-0.81)  n=20 | 0.23 (0.13-0.60)  n=18 | 0.1 (0.08-0.11)  n=12 | 0.08 (0.06-0.12)  n=13 | **< 0.001** |
| Fibrinogen level (mg/dL) *data not available in all cases | 770 (626-896) N=101 | 784 (627.3-934.8)  n=22 | 877 (792.8-1011)  n=28 | 723 (630-850)  n=30 | 592 (515.5-728)  n=21 | **< 0.001** |
| Triglycerides level (mg/dL) *data not available in all cases | 124 (99-174) N=35 | 217 (111.5-558)  n=5 | 148 (102-212)  n=7 | 120 (94.3-168)  n=10 | 113 (97.5-154)  n=13 | 0.313 |
| D-dimer level (ng/mL)  *data not available in all cases | 679.5 (431.3-1345.5)  N=98 | 1370 (649.5-3326.5)  n=22 | 755 (603.5-1118.5)  n=29 | 599 (441.8-1368)  n=26 | 345 (294-675)  n=21 | **< 0.001** |
| Monocyte count  (x 10^3^ cells/µL) | 0.4 (0.3-0.6) | 0.4 (0.2-0.6) | 0.4 (0.3-0.6) | 0.4 (0.4-0.6) | 0.4 (0.3-0.6) | 0.458 |
| Platelet count  (x 10^3^ cells/µL) | 184.5 (153.3-233.5) | 176.5 (136-229) | 216.5 (162.8-275.3) | 174 (145.3-211.5) | 212 (156.8-232.5) | 0.09 |
| Time from hospital admission to serum extraction (d), median (IQR) | 2 (2-3) | 2 (2-3) | 2.5 (2-3) | 2 (2-3) | 2 (2-3) | 0.759 |
| Length of hospital stay (d), median (IQR) | 9 (5-16) | 7.5 (5-11) | 21.5 (15.25-31) | 9.5 (6.25-15) | 5 (4-7.75) | < 0.001 |

RF: respiratory failure. ***§*** represented median and interquartile range.

**Supplementary Table 2**. Uni- and multivariate analysis of characteristics present at serum extraction predicting neutralizing antibody titer.

| Characteristics | Univariate | | | Multivariate | | |
| --- | --- | --- | --- | --- | --- | --- |
|  | Regression coefficient  B | 95% CI | *p* value | Regression coefficient  B | 95% CI | *p* value |
|  |  |  |  |  |  |  |
| Oxygen saturation | -2.389 | -5.794 – 1.016 | 0.168 |  |  |  |
| LDH | 0.168 | 0.023 – 0.314 | 0.024 |  |  |  |
| CPR | 2.141 | -0.210 – 4.492 | 0.074 |  |  |  |
| Leucocyte count | 15.393 | 7.501 – 23.285 | < 0.001 |  |  |  |
| Neutrophil count | 16.694 | 8.585 – 24.803 | 0.000 |  |  |  |
| Platelet count | 0.509 | 0.201 – 0.816 | 0.001 |  |  |  |
| GPT | 0.628 | -0.015 – 1.270 | 0.056 |  |  |  |
| Procalcitonin | 23.257 | -15.791 – 62.305 | 0.238 |  |  |  |
| Ferritin | 0.001 | -0.010 – 0.011 | 0.889 |  |  |  |
| Fibrinogen | 0.270 | 0.122 – 0.418 | 0.000 |  |  |  |
| D-Dimers | 0.000 | -0.002 – 0.002 | 0.787 |  |  |  |
| IgM | 0.013 | 0.010 – 0.016 | < 0.001 | 0.012 | 0.009 – 0.015 | < 0.001 |
| IgG1 | 0.03 | 0.017 – 0.043 | < 0.001 | 0.020 | 0.008 – 0.032 | 0.002 |
| IgG3 | 0.001 | -0.002 – 0.003 | 0.609 |  |  |  |

**Supplementary Figure 1. Relationship between antibody levels and neutralizing activity.** Levels of anti-S IgM, (A) and anti-S IgG (B), anti-S IgG1 (C) and anti-S IgG3 (D) were plotted against the neutralizing titer for the total cohort of patients (grey circles, n = 160) and the different severity groups: RF exitus (pink circles, n = 40), RF survivors (green circles, n = 40), oxygen therapy (orange circles, n = 40) and non-oxygen therapy (blue circles, n = 40). Statistical analyses were performed using Spearman’s correlation coefficient test (ρ) and *p*-value. RF indicates respiratory failure.


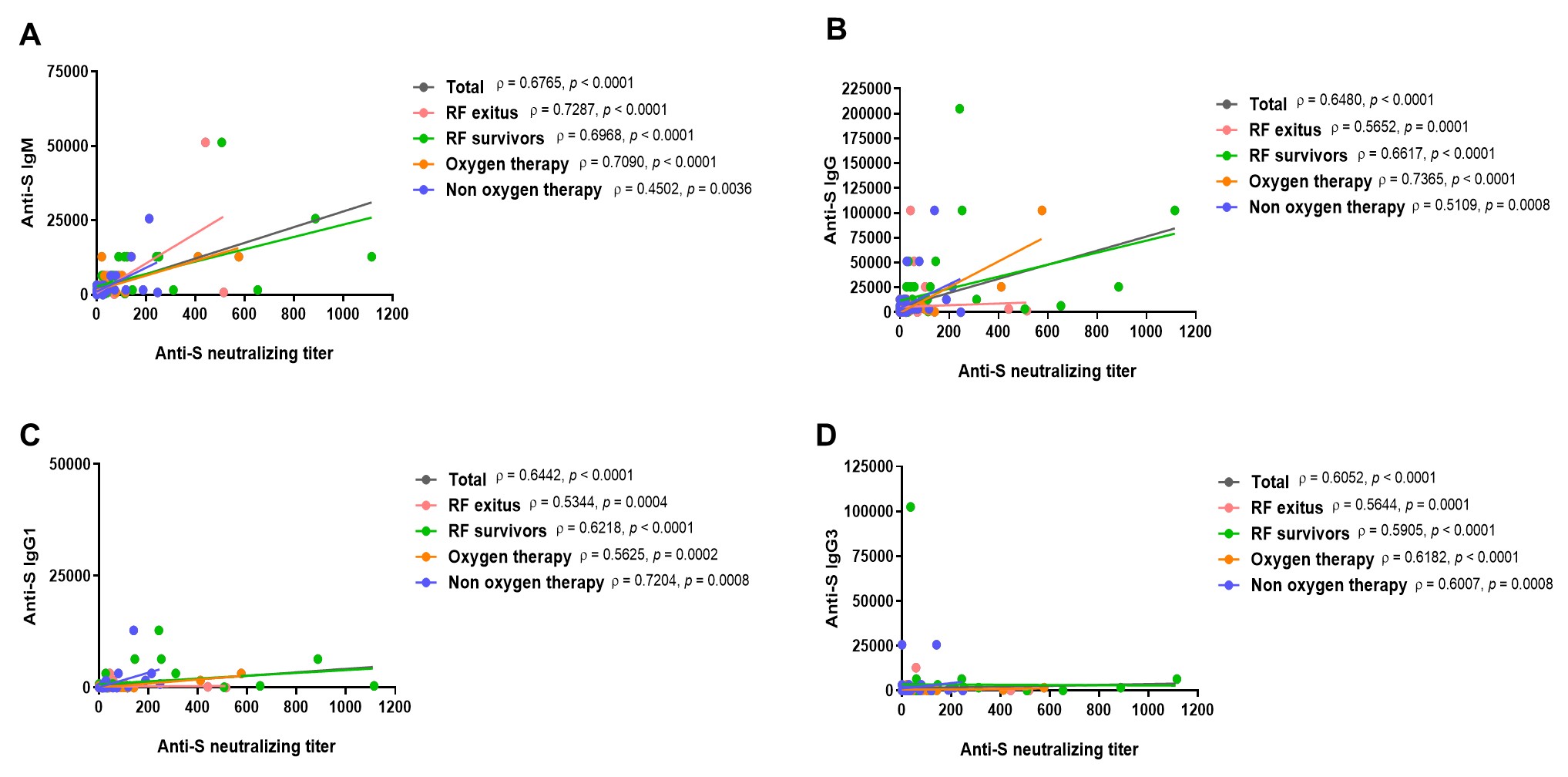


**Supplementary Figure 2. Relationship of humoral response with age, days since admission and time post-symptom onset.** Levels of anti-S IgM and anti-S IgG were plotted against the age (A, B), days since admission (C, D) and time post-symptom onset (E, F) for the total cohort of patients (grey circles, n = 160) and the different severity groups: RF exitus (pink circles, n = 40), RF survivors (green circles, n = 40), oxygen therapy (orange circles, n = 40) and non-oxygen therapy (blue circles, n = 40). Statistical analyses were performed using Spearman’s correlation coefficient test (ρ) and *p*-value. RF indicates respiratory failure.


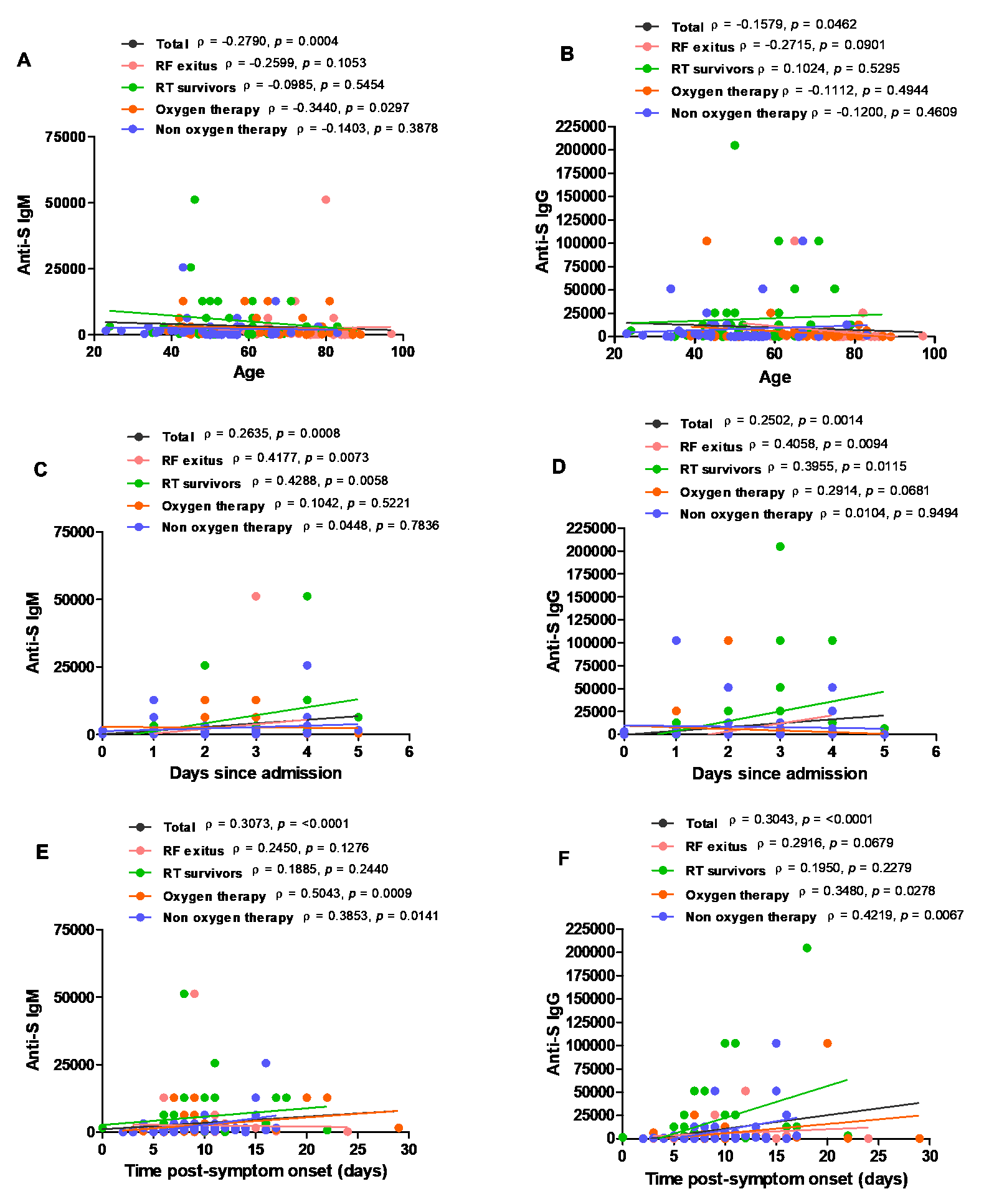

Supplement: Supplementary file 1 [file DataSheet_1.docx]
